# Beyond ejection fraction: cardiac magnetic resonance imaging in anthracycline cardiotoxicity

**DOI:** 10.1186/s12880-025-02027-y

**Published:** 2025-11-19

**Authors:** Marzieh Motevalli, Tourisa Deilami, Yasmin Mohtasham Kia, Amirhossein Poopak, Golnaz Houshmand

**Affiliations:** 1 Cardiovascular Imaging Research Center, Rajaie Cardiovascular Institute, Tehran, Iran; 2 Vascular Disease and Thrombosis Research Center, Rajaie Cardiovascular Institute, Tehran, Iran; 3 Cardio-Oncology Research Center, Rajaie Cardiovascular Institute, Tehran, Iran

**Keywords:** Anthracycline induced cardiotoxicity, Cardiac magnetic resonance imaging, Cancer therapy related cardiac disease, Cardio-oncology, Cardiac dysfunction

## Abstract

**Background:**

Anthracyclines are a cornerstone of cancer therapy, yet they carry a significant risk of cardiotoxicity, which may present as subclinical myocardial injury or overt heart failure. Timely detection is essential to prevent irreversible cardiac dysfunction and safeguard long-term quality of life. Cardiac magnetic resonance (CMR) imaging—capable of quantifying myocardial structure, function, and tissue characteristics—has emerged as a leading modality in this context. This systematic review evaluates the role of CMR in detecting both early and late cardiotoxic changes after anthracycline exposure.

**Method:**

We conducted a systematic search in accordance with PRISMA 2020 guidelines, searching PubMed, Embase, Scopus and Web of Science for studies involving CMR assessment in patients treated with anthracyclines. We extracted data on changes in functional parameters, volumetric indices, strain measurements, and tissue characterization before, during, and after anthracycline therapy in patients receiving active chemotherapy, and differences of these parameters compare to healthy adults or their pretreatment CMR scan in long term cancer survivors.

**Results:**

Across the 26 eligible studies, CMR consistently identified early declines in left ventricular ejection fraction, often before changes were detectable on echocardiography. More sensitive markers, including increases in the left ventricular end-systolic volume and alterations in strain parameters, provided early signs of subclinical dysfunction. Tissue mapping techniques revealed significant increases in native T1, T2, and extracellular volume (ECV), correlating with diffuse myocardial injury, even in the absence of late gadolinium enhancement. In long-term survivors, persistent abnormalities in strain and ECV were observed, highlighting the enduring nature of anthracycline induced cardiotoxicity. Right ventricular and left atrial remodeling, while less frequently assessed, emerged as clinically relevant and prognostically significant.

**Conclusion:**

CMR is a promising noninvasive tool that enables early detection and monitoring of anthracycline-induced cardiotoxicity, aiding risk stratification and timely cardioprotective interventions in cancer patients.

**Supplementary Information:**

The online version contains supplementary material available at 10.1186/s12880-025-02027-y.

## Introduction

 Cardiotoxicity is a serious and potentially life-threatening complication of cancer therapy, particularly with anthracycline-based regimens [1, 2]. Definitions of cardiotoxicity vary across clinical guidelines, reflecting differences in diagnostic criteria and complicating standardization [3–5]. Anthracyclines exert their cardiotoxic effects primarily through oxidative stress and direct myocardial injury, which often result in progressive and irreversible cardiac dysfunction [6, 7]. Left ventricular (LV) dysfunction and heart failure (HF) are the principal manifestations of anthracycline-induced cardiomyopathy and are strongly associated with cumulative drug exposure [8]. Vulnerability is further increased by additional risk factors such as extremes of age, female sex, African ancestry, prior chest irradiation, and pre-existing cardiovascular disease [9]. Importantly, anthracycline cardiotoxicity may arise long after therapy and can progress silently [10]. Evidence from childhood cancer survivors demonstrates that anthracycline injury is long-lasting and not strictly dose-dependent, with significant cardiac risks documented even at cumulative doses below 250 mg/m² [11]. These individuals continue to face elevated risks of heart failure and coronary artery disease for up to 45 years after treatment [12].

With global improvements in cancer survival, the early detection and prevention of anthracycline-induced cardiotoxicity (AIC) have become critical priorities [7, 13]. Effective risk stratification and early identification of myocardial structural changes may enable interventions at subclinical stages, thereby reducing long-term morbidity and improving quality of life in this growing patient population [7, 14].

Cardiac magnetic resonance imaging (CMR) has emerged as a highly sensitive, noninvasive tool for evaluating myocardial structure and function in patients at risk of anthracycline-related cardiotoxicity [3–5]. Unlike echocardiography, CMR provides comprehensive tissue characterization—detecting myocardial edema and fibrosis—together with precise volumetric, functional, and strain assessments. These capabilities highlight its value in detecting subtle impairments in cardiac performance before overt clinical manifestations develop.

Despite increasing interest in CMR for cardiotoxicity monitoring, important knowledge gaps remain regarding its standardized application, optimal imaging protocols, and integration into routine follow-up. Existing studies differ considerably in patient selection, chemotherapy regimens, and imaging parameters, limiting comparability. Moreover, definitive evidence linking specific CMR indices to long-term outcomes is still scarce.

This systematic review therefore aims to consolidate current knowledge on the role of CMR in detecting anthracycline-induced cardiotoxicity. By critically appraising existing clinical research, we highlight the prognostic value of CMR indices, identify methodological inconsistencies, and assess whether imaging findings reliably predict adverse cardiac events. Clarifying both the potential and the limitations of CMR in this field may inform clinical decision-making and guide future strategies for surveillance.

## Methods

This systematic review and meta-analysis followed the Preferred Reporting Items for Systematic Reviews and Meta-Analyses (PRISMA) 2020 guidelines [15]. The protocol was prospectively registered in the PROSPERO database (registration code: CRD420251027587).

### Search strategy

We conducted a comprehensive systematic search across four major online databases, PubMed, Embase, Scopus and Web of science. The search strategy was developed by a single reviewer (YMK) using the following keywords: (“Cardiac Magnetic Resonance Imaging” OR “Cardiovascular Magnetic Resonance”) AND (“Chemotherapy Induced Cardiomyopathy” OR “Myocardial Damage” OR “Cardiotoxicity” OR “Cardiac dysfunction”) AND (“Anthracycline*”). The full search strategy is detailed in Table 1 (available in the supplementary file). We also screened the references of relevant studies to identify eligible studies.

### Study selection

Duplicates were removed using EndNote^®^. Two reviewers (YMK, AHP) independently screened titles and abstracts, resolving disagreements through discussion; a third reviewer (MM) was consulted when necessary. Studies were selected according to predefined PICO criteria (population, intervention, comparator, outcome, and study design) and the inclusion/exclusion rules outlined below.

We used the PICO framework as follows:

- Patient/Population (P): Patients with cancer who received anthracycline based regimen.
- Intervention (I): Cardiac MRI.
- Compare (C): Baseline and follow up (early and late) CMRI indices.
- Outcome (O): CMRI prognostic value in anthracycline included chemotherapy regimen.

Inclusion criteria:

- Cancer patients (including pediatric and adult patients) actively receiving anthracycline treatment with or without trastuzumab.
- Long term cancer survivors (at least a median of 11 months from the last chemotherapy session) who had received anthracyclines containing regimen with or without trastuzumab.
- Patients without a history of baseline cardiovascular disease, including cardiomyopathies (ischemic or non-ischemic), heart failure, congenital heart disease, coronary artery disease, valvular heart disease, etc.
- Human studies only.
- Study designs limited to randomized controlled trials (RCTs), prospective or retrospective cohort studies, and cross-sectional studies.

Exclusion criteria:

- Non-anthracycline regimens.
- Use of cardioprotective agents (e.g., dexrazoxane).
- Case reports, editorials, letters, abstracts, animal studies, and non-English publications.

### Data extraction

Data extraction was performed independently by two reviewers (YMK and TD). Information collected included: first author, year of publication, study design, sample size, cancer type, chemotherapy regimen, CMR field strength, follow-up intervals for imaging, and main study outcomes.

## Results

**Fig. 1:**
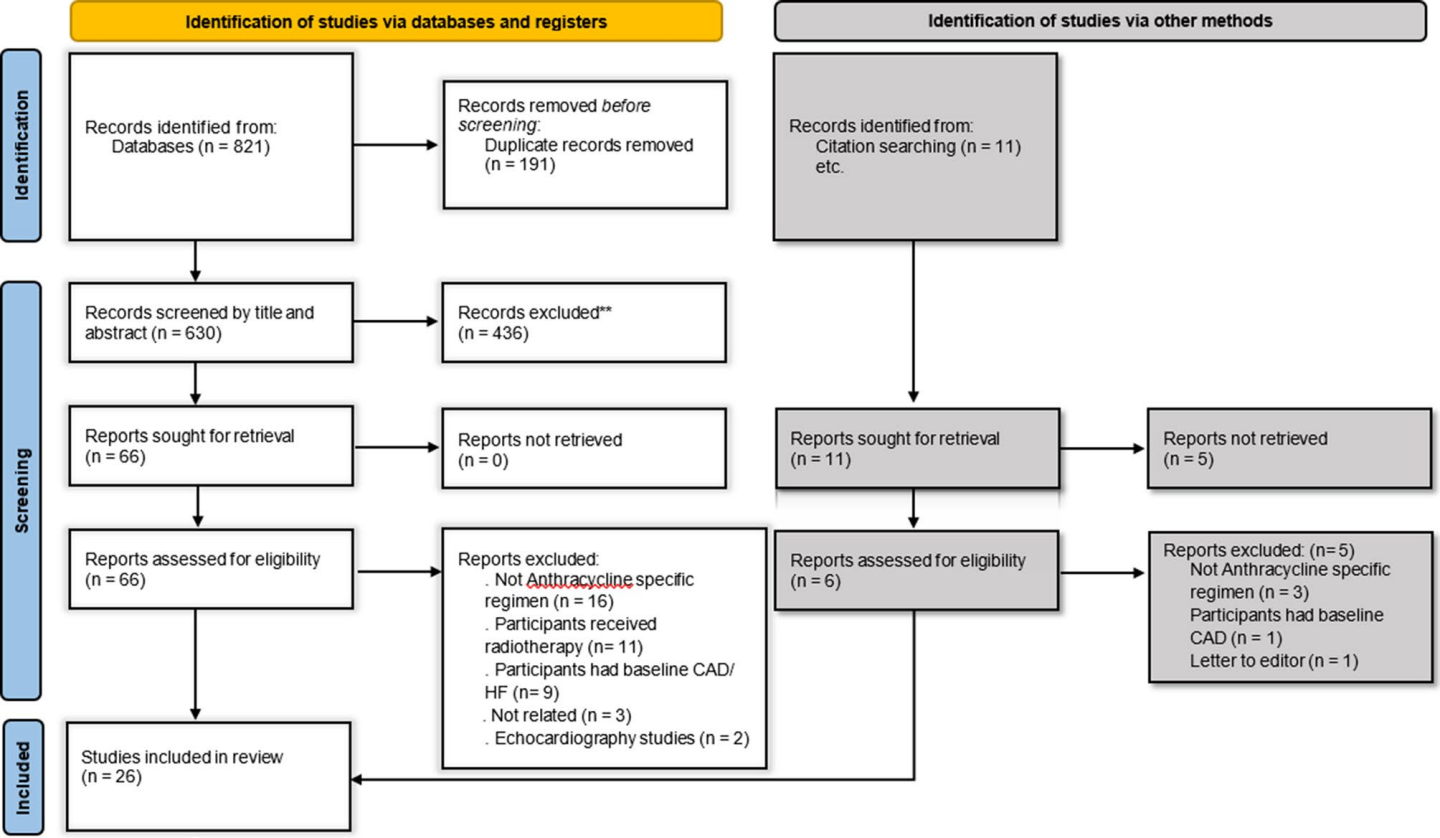
PRISMA flow chart summarizing the selection process of eligible studies

The initial search yielded 821 records, 59 from PubMed, 589 from Embase, 64 from Scopus, and 109 from Web of science. After removing duplicates, 630 studies remained. Title-Abstract screening resulted in 64 studies which after full-test screening 25 remained. We included one study from manual citation searching and finally included 26 eligible studies for our systematic review. A detailed flowchart of searching and exclusion reasons is demonstrated in Fig. 1.

The important characteristics of all the 26 included studies are stated in Tables 2 and 3.

It is noteworthy that studies that stated their participants received radiotherapy or other chemotherapy regimen than anthracycline (± trastuzumab) and studies which included patients with baseline cardiovascular disease were all excluded.

### CMR findings

#### Early anthracycline-induced myocardial alteration

##### Functional parameters

LVEF was the most frequently reported CMR-derived parameter, assessed at baseline and after anthracycline therapy. Nearly all studies documented a decline in LVEF during treatment [16–18]. Tak et al. reported a significant decrease in LVEF (*p* = 0.009) after doxorubicin (mean dose 459.3 ± 92.5 mg/m²) in 18 cancer patients, whereas echocardiography performed concurrently did not detect this change [19]. In a prospective study of 30 breast cancer patients (15 with CTRCD and 15 without), both groups showed a significant reduction in LVEF at 3 and 6 months, with the decline more pronounced in the CTRCD-positive group [20]. Wassmuth et al. found a modest but significant LVEF decrease (from 67.8% ± 1.4% to 58.9% ± 1.9%, *p* < 0.05) in 22 patients 28 days after chemotherapy [21]. Grover et al. reported significant reductions in LVEF at 4 months (*p* = 0.001) and 14 months (*p* = 0.001) in breast cancer patients [22]. In contrast, Lustberg et al. observed no significant early LVEF change 2–4 weeks post-therapy (*p* = 0.30), but a significant decline (− 7.5 ± 1.8%, *p* < 0.001) was evident at a median 19.8 months follow-up [23].

**Table 2:** Summary of studies on early anthracycline-induced myocardial alterations

Author	Year	Design	*N*	Cancer type	Chemo regimen	CMRI	CMR interval	Outcomes
Ibrahim et al. [54]	2025	Prospective	9	Breast cancer	Anthracycline	3 T	Baseline, post-treatment, 6 m FU	-LVEF and RVEF decreased post-treatment, then recovered at 6 m FU. -T1, T2, ECV continuously increased -The 6 m FU strain was slightly higher than the post-treatment strain, especially for GCS and GRS.
Li et al. [29]	2024	Prospective	82	Breast cancer	Anthracycline ± Trastuzumab	3 T	Baseline, 3 m, 6 m	-There was a significant LVEF reduction. -No significant change in LVESVi, LVEDVi, LV mass index during FU in CRTCD + group. -Strain measurements decreased, with GLS showing a significant reduction in CRTCD+. -T1ρ, T1, and T2 exhibited an increase from baseline to 6 months.
Thavendiranathan et al. [30]	2023	Prospective	136	Breast cancer	Anthracycline ± Trastuzumab	1.5 T	Baseline, after anthracycline completion (53 days from baseline), 3 m, 6 m, post trastuzumab	-GLS/GCS, or LV mass index peaked after anthracycline treatment and then recovered - None developed new LGE -T1, T2, and ECV increased.
Thavendiranathan et al. [24]	2023	RCT	112	Cancer patients (Breast cancer, Lymphoma, Leukemia, Sarcoma, or Thymoma)	Anthracycline	1.5 T	Baseline, median 22 days after anthracycline treatment completion	-There was a significant decrease in LVEF and a significant LVESV increase. -GLS and GCS worsened. -None developed LGE.
P.Houbis et al. [25]	2021	Prospective	125	Breast cancer	Anthracycline ± Trastuzumab	1.5 T	Baseline, 3 m, 6 m, 9 m, 15 m	**-**Over FU period there was a significant decline in LVEF, significant increase in LVESVi, LVEDVi which were assoiciated with subsequent CTRCD. **-**Tagged-CMR GLS/GCS and FT-GCS were early predictors of subsequent CTRCD.
Ferreira de Souza et al. [28]	2021	Prospective (post-Hoc)	27	Breast cancer	Anthracycline	3 T	Baseline, 3 consecutive visits after anthracycline (~ 140, 231, and 427 days from baseline)	-No change in RVEDVi. -RVESVi and RV ECV had increased significantly. -RVEF and RV mass index declined.
Lambert et al. [20]	2019	Prospective	60	Breast cancer	Anthracycline ± Trastuzumab	1.5 T	Baseline, 3 m, 6 m	-A significant reduction in LVEF and GLS was seen in both CTRCD + and CTRCD-.
A. Altaha et al. [18]	2019	Prospective	50	Breast cancer	Anthracycline	1.5 T	Baseline, 3 m, 6 m	-CTRCD + had a significant reduction in LVEF and GLS (> 15% change) -CTRCD + had a significant increase in native T1, T2 and ECV.
Lustberg et al. [55]	2019	Prospective	29	Breast cancer	Anthracycline	1.5 T	Baseline, after 1st chemo cycle, 2–4 w after last chemo cycle	-LVEF and GCS remained unchanged, while T2 increased progressively during chemotherapy.
Tak et al. [19]	2019	Prospective	18	Breast cancer Lymphoma	Anthracycline	1.5 T	Baseline, after 4 cycles of chemotherapy	-LVEF significantly decreased from baseline to post treatment.
Ferreira de Souza et al. [16]	2018	Prospective	27	Breast cancer	Anthracycline	3 T	Baseline, 3 consecutive visits after anthracycline (~ 140, 231, and 427 days from baseline)	-LVEF, LV mass index and LV mass-to-volume ratio significantly decreased. -There was a significant increase in the T2 during the first 3 m. -ECV increased during FU.
Toro-Salazar et al. [17]	2018	Prospective	13	Cancer patients (ALL, AML, HL, Osteosarcoma)	Anthracycline	1.5 T	Baseline, 24–48 h after anthracycline cumulative dose, at maximal dose, 1y after treatment	-LVEF decreased significantly. -High anthracycline doses reduced GLS and GCS, whereas low doses did not significantly affect GCS.
Avelar et al. [26]	2017	Prospective	20	Breast cancer	Anthracycline ± Trastuzumab	1.5 T	Baseline, during chemotherapy, 2w after chemotherapy, and 6 m after chemotherapy)	-LVEF decreased significantly. -LVEDV and LVESV significantly increased. -Relative wall thickness decreased significantly. -No LGE was detected.
De Ville de Goyet et al. [27]	2015	Prospective	81	Children with cancer (ALL, AML, HL, NHL, Sarcoma, NB, Brain tumor, RB, Nephroblastoma, HBL, MDS	Anthracycline	1.5 T	. One and two years after chemotherapy initiation	-There was a significant increase in RVEF and RV mass and a decrease in RVESV. -LVEDV significantly increases from base line to 2y FU. -Left atrial volume significantly increased.
Grover et al. [22]	2015	Prospective	29	Breast cancer	Anthracycline ± Trastuzumab	1.5 T	Baseline, 1 m, 4 m and 14 m post-chemotherapy	-LVEF significantly decreased (after 4 m and also 14 m).
Wassmuth et al. [21]	2001	Prospective	22	Breast cancer, NHL, Osteosarcoma, HL, primitive neuroectodermal tumor, LMS, chondrosarcoma, and endometrial carcinoma	Anthracycline	1 T	Baseline, 3 days and 28 days after anthracycline chemotherapy	- LVEF showed a slight but significant decrease. -Relative myocardial contrast enhancement increased significantly.

ALL = Acute Lymphoblastic Leukemia, AML = Acute Myeloblastic Leukemia, CMRI = cardiac magnetic resonance imaging, CTRCD = cancer therapy related cardiac dysfunction, ECV = extracellular volume fraction, FT = feature tracking, FU = follow-up, GCS = global circumferential strain, GLS = global longitudinal strain, GRS = global radial strain, HBL = Hepatoblastoma, HL = Hodgkin Lymphoma, LMS = Leiomyosarcoma, LV = left ventricle, LVEF = left ventricular ejection fraction, LVEDVi = left ventricular end diastolic volume index, LVESVi = left ventricular end systolic volume index, m = month(s), MACE = major adverse cardiac events, MDS = Myelodysplasia, NB = Neuroblastoma, NHL = Non-Hodgkin Lymphoma, RB = Retinoblastoma, RMS = Rhabdomyosarcoma, RV = right ventricle, RVEDVi = right ventricular end diastolic volume index, RVESVi = right ventricular end systolic volume index, w = week(s)

**Table 3:** Summary of studies on late anthracycline-induced myocardial alterations

Author	Year	Design	*N*	Cancer type	Chemo regimen	CMRI	CMR interval	Outcome
Chhikara et al. [38]	2022	retrospective	249	Cancer survivors (Breast cancer, Lymphoma, Leukemia, or Sarcoma)	Anthracycline ± Trastuzumab	1.5 T	Median of 2.9y after chemotherapy	-The prevalence of LV dysfunction was 69.9%. -RV systolic dysfunction was present in 21.7% of patients, 92.6% of whom also had LV dysfunction. -Reduced RVEF was associated with MACE.
Harries et al. [37]	2021	Prospective	44	Cancer survivors (Breast cancer, Hematological), Healthy controls	Anthracycline	1.5 T	Median of 11 months after chemotherapy completion	- Cancer survivors had lower LV mass index, inversely correlated with anthracycline dose. -T1 and ECV were significantly higher in survivors. -GLS was significantly impaired in survivors. -LVESVi, LVEDVi and LVEF were similar between both groups. -RVESVi, RVEDVi and RVEF were similar between both groups.
Wolf et al. [40]	2020	Retrospective	79	Cancer survivors (ALL, HL, Sarcoma, Nephroblastoma, NB, HBL)	Anthracycline	1.5 T	11.2 ± 4.5 y after cancer treatment	-No abnormality in T1, ECV and LGE was found among survivors.
Mokshagundam et al. [39]	2020	Prospective (pilot)	30	Childhood cancer survivors (Osteosarcoma, Ewing’s sarcoma, ALL, AML, Lymphoma, Wilms tumor, RMS)	Anthracycline	1.5 T	Median of 32.6 m after chemotherapy completion	-ECV was significantly correlated with cumulative anthracycline dose.
M. Long et al. [56]	2019	Prospective	22	Childhood leukemia survivors	Anthracycline	1.5 T	≥ 5 years after chemotherapy	-LVEF was significantly reduced in survivors. - No difference in RVEF or LVEDV between groups.
Kimball et al. [33]	2018	Prospective	26	Cancer survivor (breast cancer)	Anthracycline ± Trastuzumab	na	5 ± 1 years post chemotherapy	- LVEF decreased but without clinical significance -T1 values were within normal limits. -There was a significant increase in LVESV. - RVEDV and RVESV significantly declined after 5-year FU.
Toro-Salazar et al. [44]	2015	Prospective	57	Childhood cancer survivors (Leukemia, Solid tumor, Lymphoma)	Anthracycline	1.5 T	2.4 to 26.9 years after chemotherapy	-Survivors showed significantly lower midwall peak circumferential strain and lower midwall peak longitudinal strain.
Ylänen et al. [34]	2014	Prospective	71 (total) 58 (CMR)	long-term survivors of childhood cancer	anthracycline	na	5 < years after cancer survival	-Survivors had larger LVESV and lower LVEF -None showed LGE.
Tham et al. [36]	2013	Prospective	30	Cancer survivors (ALL, Lymphoma, AML, Wilms tumor, Ewings sarcoma, NB, Osteosarcoma, hemangiopericytoma)	Anthracycline	1.5 T	at least 2 years following chemotherapy	-No LGE was found. -ECV showed significant correlations with anthracycline dose. - Baseline myocardial T1 was higher in those receiving larger chemotherapy doses. -CMR global function and mass were normal
G.Neilan et al. [41]	2012	Retrospective	91	Cancer survivors with anthracycline induced cardiomyopathy	Anthracycline	1.5 T or 3 T	Median of 88 m after chemotherapy	-Lower LV mass index with higher AC dose; predicted MACE.

ALL = Acute Lymphoblastic Leukemia, AML = Acute Myeloblastic Leukemia, CMRI = cardiac magnetic resonance imaging, CTRCD = cancer therapy related cardiac dysfunction, ECV = extracellular volume fraction, FT = feature tracking, FU = follow-up, GCS = global circumferential strain, GLS = global longitudinal strain, GRS = global radial strain, HBL = Hepatoblastoma, HL = Hodgkin Lymphoma, LMS = Leiomyosarcoma, LV = left ventricle, LVEF = left ventricular ejection fraction, LVEDVi = left ventricular end diastolic volume index, LVESVi = left ventricular end systolic volume index, m = month(s), MACE = major adverse cardiac events, MDS = Myelodysplasia, NB = Neuroblastoma, NHL = Non-Hodgkin Lymphoma, RB = Retinoblastoma, RMS = Rhabdomyosarcoma, RV = right ventricle, RVEDVi = right ventricular end diastolic volume index, RVESVi = right ventricular end systolic volume index, w = week(s)

##### Volumetric parameters

Thavendiranathan et al. reported increases in LVEDV and LVESV within 4 weeks of therapy, with LVESV reaching significance (*p* < 0.001) [24]. Houbois et al. found increases in LV volumes and showed that adding LVESV improved prediction of CTRCD [25]. Avelar et al. demonstrated significant increases in LVEDV and LVESV during treatment [26]. Pediatric cohorts showed LVEDV rise and left atrial enlargement linked to dose and future diastolic dysfunction [27]. Ferreira de Souza et al. noted RVESV increase with RVEF decline after therapy [28].

##### Tissue characterization

Quantitative mapping revealed increases in native T1, T2, and ECV during or shortly after therapy [29, 30]. Li et al. found T1ρ mapping improved prediction of CTRCD, with increases preceding LVEF decline [29]. Thavendiranathan et al. confirmed temporal rises in T1, T2, and ECV, with ECV predicting later CTRCD [30]. Altaha et al. showed greater changes in CTRCD-positive versus negative patients [18]. No new LGE was detected [24, 26, 30].

Early increases in native T1 and ECV have also been shown to predict subsequent cancer therapy-related cardiac dysfunction; in one study, native T1 at first follow-up had an AUC of ~ 0.71, which improved when combined with LVEF [31].

##### Feature tracking

CMR-FT strain showed early abnormalities. Toro-Salazar et al. demonstrated significant reductions in GLS and GCS at high doses, preceding LVEF decline [17]. Houbois et al. linked GCS changes with future CTRCD [25]. GLS declined significantly in CTRCD-positive breast cancer patients during 6 months of follow-up [20]. Li et al. observed GLS reduction in CTRCD-positive patients at 3 and 6 months, with GCS and GRS also significantly reduced in both CTRCD-positive and negative groups [29].

#### Late anthracycline-induced myocardial alteration

Cardiomyocyte injury persists long after therapy. Multiple studies reported reduced LVEF and elevated LVESV years after treatment, with greater abnormalities in childhood cancer survivors [32–35]. Harries et al. found no difference between survivors and controls at 11 months post-therapy [36], while a larger cohort showed RV dysfunction predicting MACE [37]. Tissue markers such as T1 and ECV were elevated in survivors [36, 38], though some long-term studies showed no differences [39]. Myocardial edema was uncommon [40]. Strain studies confirmed impaired GLS and circumferential/longitudinal strain in survivors compared with controls [36, 41].

Other CMR parameters beyond ventricular volumes and standard EF have prognostic significance. For example, Neilan et al. found that indexed left ventricular mass by CMR was a strong independent predictor of adverse cardiovascular outcomes (heart failure hospitalization and death) in patients with anthracycline cardiomyopathy, even after adjustment for anthracycline dose and EF [41].

Late gadolinium enhancement (LGE) was infrequently observed in long-term anthracycline cohorts, with most studies reporting absent or only minimal focal LGE despite persistent abnormalities in mapping and strain [42]. Although LGE, when present, likely reflects focal replacement fibrosis and has prognostic value in non-ischemic cardiomyopathies, the prevalence and prognostic role of LGE specifically after anthracycline exposure remains limited and inconsistent across studies [43]. Therefore, multiparametric mapping (native T1 and ECV) may be a more sensitive marker of anthracycline-related myocardial injury than LGE alone [5].

## Discussion

**Fig. 2:**
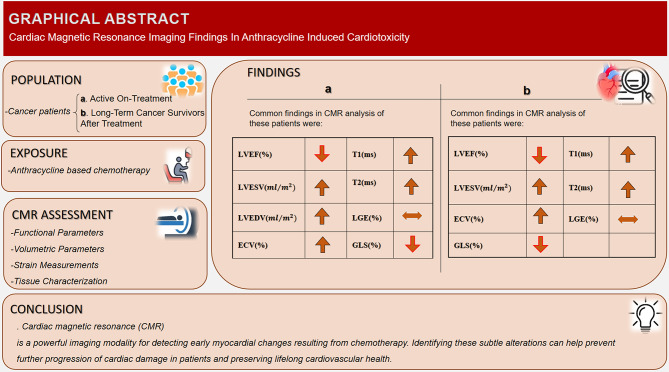
Summary of Cardiac Magnetic Resonance Findings in Anthracycline-Induced Cardiotoxicity. This figure provides an overview of the study population, CMR assessment parameters, and characteristic imaging findings in patients with anthracycline-induced cardiotoxicity. **Panel (a)** illustrates early CMR changes observed during active anthracycline therapy, including reduced LVEF and GLS, alongside increased LVESV, T1, T2, ECV. **Panel (b)** summarizes late findings in long-term cancer survivors following anthracycline exposure, demonstrating persistent reduction in GLS and LVEF, elevated ECV and T1 values reflecting residual interstitial fibrosis, and variable changes in LGE. These findings highlight the chronic and progressive nature of anthracycline-related myocardial remodeling detectable by CMR. **a**: Active anthracycline treatment. **b**: Long-Term cancer survivors. increased . decreased . variable results . ECV, extracellular volume fraction; GLS, global longitudinal strain; LGE, late gadulinium enhancement; LVEDV, left ventricular end diastolic volume; LVEF, left ventricular ejection fraction; LVESV, left ventricular end systolic volume

Anthracyclines remain central to modern oncology [44], but their associated risk of cancer-therapy-related cardiac dysfunction presents a growing challenge [45]. By synthesizing 25 studies, including one RCT, this review provides a comprehensive overview of anthracycline cardiotoxicity assessed by CMR. Three conclusions emerge: (i) CMR detects functional decline earlier than echocardiography; (ii) multiparametric tissue characterization and strain reveal preclinical injury; and (iii) RV remodeling provides additional prognostic value (Fig. 2). These findings are consistent with current ESC cardio-oncology guidelines, which recommend CMR as a complementary tool while recognizing that widespread implementation is limited by cost and availability [46].

### Early functional decline

LVEF decreased by 3–12% within 12 months of therapy, with nadirs at 4–14 months [46]. Echocardiography often underestimated these changes. LVESV increases were more sensitive, improving prediction of CTRCD [25], supporting recommendations for both fractional and volumetric monitoring [47].

### Multiparametric tissue characterization

CMR mapping detected early increases in T1 (9–23 ms), T2 (0.7–2.0 ms) and ECV (1.6–4.0%), preceding LVEF decline. T1 changes predicted CTRCD [29]. Absence of LGE indicated diffuse rather than focal fibrosis, emphasizing the value of mapping.

### Strain imaging

CMR-FT strain consistently showed early deterioration in GLS and GCS, predictive of CTRCD [48]. Compared with echocardiographic strain, CMR-FT demonstrated better reproducibility, particularly in technically challenging patients [49].

### RV and atrial remodeling

RV dysfunction predicted MACE independent of LV indices [37]. LA enlargement correlated with anthracycline dose and predicted diastolic dysfunction in children. Routine biventricular and atrial analysis is warranted.

### Survivors

Even decades after therapy, survivors showed abnormal strain, higher LVESV, reduced LV mass, and elevated ECV/T1 despite preserved LVEF [37, 38, 50]. LGE remained uncommon. These findings support long-term CMR follow-up at 2–5 year intervals in high-risk survivors.

### Integration with practice

Current cardio-oncology guidelines recommend baseline cardiovascular risk assessment and individualized imaging surveillance for patients receiving potentially cardiotoxic therapy. Echocardiography remains the first-line modality for most patients because of its accessibility and cost-effectiveness, while CMR should be reserved for specific clinical scenarios where it provides incremental value [51].

High-risk patients include those receiving high cumulative anthracycline doses, prior or planned mediastinal radiotherapy, extremes of age, pre-existing cardiovascular disease or reduced baseline LVEF, multiple cardiovascular risk factors, concomitant cardiotoxic agents (e.g., trastuzumab), or abnormal baseline biomarkers (troponin or natriuretic peptides). In these groups, guidelines recommend a baseline CMR when echocardiographic image quality is suboptimal, when precise quantification of function or tissue characterization may affect management, or when prior cardiotoxic exposure is present [52].

To avoid overburdening healthcare resources, a tiered imaging strategy is proposed [4, 51, 53]:

- All patients: baseline TTE and biomarkers before chemotherapy.
- High-risk or equivocal cases: baseline CMR including cine imaging, feature-tracking strain, native T1/T2 mapping, and, when feasible, post-contrast T1 mapping with ECV and LGE imaging.
- Follow-up: repeat imaging during therapy and post-treatment; CMR is indicated for unexplained LV dysfunction, discordant imaging results, or persistent biomarker elevation. When contrast use is contraindicated, a non-contrast CMR protocol including cine imaging and native T1/T2 mapping still provides valuable diagnostic information.

These recommendations align with recent ESC and ASE position statements advocating risk-adapted, multiparametric CMR for surveillance of anthracycline-induced cardiotoxicity. A pragmatic approach can optimize detection of cardiotoxicity without overwhelming imaging capacity or healthcare budgets [5].

### Strengths and limitations

This review applied PRISMA methodology and included both pediatric and adult populations. Limitations include heterogeneity in imaging protocols, including both pediatric and adult population, lack of pooled meta-analysis, reliance on observational studies, and predominance of breast cancer cohorts. Moreover, another major limitation of the available literature is the paucity of long-term outcome data linking CMR-derived imaging biomarkers with hard clinical endpoints such as heart failure, hospitalization, or mortality. Consequently, while these imaging parameters are promising, their direct impact on patient management and outcomes remains to be fully established. Most included studies had relatively small sample sizes, which may limit the generalizability of our findings and reduce the statistical power of pooled interpretations.

## Conclusion

While echocardiography remains the first-line imaging modality for surveillance, CMR serves as a valuable complementary tool for accurate quantification and tissue characterization, particularly in high-risk patients or when echocardiographic findings are inconclusive. As cancer survivorship rises, collaboration between cardiology and radiology, informed by high-quality CMR evidence, will be vital to protecting long-term cardiovascular health.

## Supplementary Information

Below is the link to the electronic supplementary material.

Supplementary Material 1

## Data Availability

Data sharing is not applicable to this article as no datasets were generated or analysed during the current study.

## Data availability

Data sharing is not applicable to this article as no datasets were generated or analysed during the current study.

## Abbreviations

CMRI: Cardiac magnetic resonance imaging
CMR: Cardiac magnetic resonance
CTRCD: Cancer therapy related cardiac dysfunction
AIC: Anthracycline induced cardiotoxicity
HF: Heart failure
LV: Left ventricle
LVEF: Left ventricular ejection fraction
RV: Right ventricle
RVEF: Right ventricular ejection fraction
LVESV: Left ventricular end systolic volume
LVEDV: Left ventricular end diastolic volume
RVESV: Right ventricular end systolic volume
RVEDV: Right ventricular end diastolic volume
ECV: Extracellular volume
LGE: Late gadolinium enhancement
GLS: Global longitudinal strain
GCS: Global circumferential strain
GRS: Global radial strain
