# Supplementary material for: Beyond ejection fraction: cardiac magnetic resonance imaging in anthracycline cardiotoxicity
Source: BMC Med Imaging. 2025 Nov 19;25:478. doi: 10.1186/s12880-025-02027-y (PMC12628630; doi:10.1186/s12880-025-02027-y)
Supplement: Supplementary file 1 — Supplementary Material 1 [file 12880_2025_2027_MOESM1_ESM.docx]

**The role of cardiac magnetic resonance imaging in the detection of anthracycline related cardiotoxicity: a systematic review**

Search strategy

Table.1

| **Pubmed** | **Query** | **Results** |
| --- | --- | --- |
| **#1** | "Cardiac Magnetic Resonance Imaging"[tiab] | 9468 |
| **#2** | "Ventricular Dysfunction"[Mesh] OR "Ventricular Dysfunction, Right"[Mesh]" OR “Ventricular Dysfunction, Left"[Mesh]” OR "chemotherapy induced cardiomyopathy"[tiab] OR "myocardial damage"[tiab] OR “cardiotoxicity”[tiab] OR “cardiac dysfunction”[tiab] OR “systolic dysfunction”[tiab] OR “diastolic dysfunction”[tiab] | 62401 |
| **#3** | "Anthracyclines"[Mesh] OR "Anthracycline*"[tiab] | 91005 |
| **#1 AND #2 AND #3** |  | 59 |
| **Embase** | **Query** | **Results** |
| **#1** | (‘cardiovascular magnetic resonance’/exp OR ‘cardiac magnetic resonance’:ab,ti OR ‘cardiac magnetic resonance imaging’:ab,ti OR ‘cardiac MRI’:ab,ti OR ‘cardiovascular magnetic resonance imaging’:ab,ti OR ‘cardiovascular MRI’:ab,ti OR ‘CMR (cardiovascular magnetic resonance)’:ab,ti OR ‘cardiovascular magnetic resonance’:ab,ti) | 84101 |
| **#2** | (‘chemotherapy induced cardiomyopathy’/exp OR ‘ventricular dysfunction’/exp OR ‘left ventricle dysfunction’/exp OR ‘right ventricle dysfunction’/exp OR ‘left ventricular function’/exp OR ‘right ventricular function’/exp OR ‘Systolic dysfunction ‘/exp OR ‘Diastolic dysfunction’/exp OR ‘ventricular dysfunction’:ab,ti OR ‘left ventricle dysfunction’:ab,ti OR ‘right ventricle dysfunction’:ab,ti OR ‘left ventricular function’:ab,ti OR ‘right ventricular function’:ab,ti OR ‘Systolic dysfunction ‘:ab,ti OR ‘Diastolic dysfunction’:ab,ti OR ‘chemotherapy induced cardiomyopathy’:ab,ti OR ‘cardiotoxicity’/exp OR ‘cardiotoxicity’:ab,ti) | 231929 |
| **#3** | ‘anthracycline’/exp OR ‘anthracycline’:ab,ti) | 41259 |
| **#1 AND #2 AND #3** |  | 589 |
| **Scopus** | **Query** | **Results** |
| **#1** | (TITLE-ABS (“Cardiac Magnetic Resonance Imaging" OR “cardiac MRI” OR “cardiovascular magnetic resonance imaging” OR “cardiovascular MRI”)) | 16332 |
| **#2** | (TITLE-ABS ("Ventricular Dysfunction" OR “cardiac dysfunction” OR “systolic dysfunction” OR “diastolic dysfunction” OR “chemotherapy induced cardiomyopathy” OR “cardiotoxicity”)) | 72243 |
| **#3** | (TITLE-ABS ("anthracycline”)) | 20163 |
| **#1 AND #2 AND #3** |  | 64 |
| **WOS** | **Query** | **Results** |
| **#1** | (TS= (“Cardiac Magnetic Resonance Imaging" OR “cardiac MRI” OR “cardiovascular magnetic resonance imaging” OR “cardiovascular MRI”)) | 19629 |
| **#2** | (TS= ("Ventricular Dysfunction" OR “cardiac dysfunction” OR “systolic dysfunction” OR “diastolic dysfunction” OR “chemotherapy induced cardiomyopathy” OR “cardiotoxicity”)) | 103307 |
| **#3** | (TS= ("anthracycline”)) | 18349 |
| **#1 AND #2 AND #3** |  | 109 |

Table 1. The search strategy based on key words in 4 database including PubMed, Embase, Scopus and Web Of Science.
